# Is the Water Supply a Key Factor in Stingless Bees’ Intoxication?

**DOI:** 10.1093/jisesa/ieaa127

**Published:** 2020-11-12

**Authors:** Annelise de Souza Rosa-Fontana, Adna Suelen Dorigo, Hellen Maria Soares-Lima, Roberta Cornélio Ferreira Nocelli, Osmar Malaspina

**Affiliations:** 1 Centro de Estudos de Insetos Sociais – CEIS, Instituto de Biociências, Universidade Estadual Paulista Júlio de Mesquita Filho (UNESP-SP), Rio Claro, SP, Brasil; 2 Departamento de Ciências da Natureza, Matemática e Educação, Universidade Federal de São Carlos (UFSCar-SP), Araras, SP, Brasil

**Keywords:** pollinator, imidacloprid, field realistic concentration, Brazilian bees

## Abstract

Water is an important resource for stingless bees, serving for both honey dilution and the composition of larval food inside nests, yet can be an important route of exposure to pesticides. Assuming bees can forage naturally on pesticide-contaminated or noncontaminated areas, we investigated whether water supply influences the choice between neonicotinoid-dosed or nondosed feeders and on mortality of the stingless bee, *Melipona scutellaris* (Latreille, Hymenoptera, Apidae). At the field concentration, there was no significant mortality; however, the bees were not able to distinguish the feeders. In the cages containing high-concentration feeders, with water supply, the bees preferred nondosed food, and with no water, the mortality increased. Considering that in the field it is common to find extrapolated concentrations, our work suggested that water may allow avoidance of high dosed food and minimize mortality.

Neonicotinoids have been linked to pollinator losses by impairing the ability for foraging, feeding, and learning behavior (Decourtye et al. 2004a,b; Henry et al. 2012; Schneider et al. 2012). These effects in combination may lead to colony failure (Gill et al. 2012, Laycock et al. 2012, Whitehorn et al. 2012, Lu et al. 2020). Based on the risk assessment of the European Food Safety Authority (EFSA) in 2012, the commission’s members of the European Union severely restricted the use of plant protection products and treated seeds containing three neonicotinoids (clothianidin, imidacloprid, and thiamethoxam) to protect honeybees in 2013. Nevertheless, concerning for other important pollinators, such as bumblebees, generated additional assessments using the same approach (Cresswell et al. 2012, Goulson 2013, Feltham et al. 2014, Kessler et al. 2015, Thompson et al. 2015). This issue is a worldwide concern, including concerns about potential effects on native pollinators (de Souza Rosa et al. 2015, Hladik et al. 2016), including in Brazil.

Currently, many countries use honeybees as a surrogate to evaluate the risk of pesticides to all species of bees. However, the extent to which honeybees can serve as surrogates for non-*Apis* bee species is questionable. Regarding the Neotropical area, Cham et al. (2019) pointed out several life-history traits of stingless bees, which stingless bee biology and pesticide exposure routes are not covered by the current honey bee exposure assessment paradigm. Among the several particularities, water is an important resource for stingless bees, since it will serve for both honey dilution and the composition of larval food (Hartfelder and Engels 1989, Roubik 2006).

Recent advances in the scientific literature provide several investigations of the pesticide effects on stingless bees (Bernardes et al. 2018, Boff et al. 2018, de Morais et al. 2018, Moreira et al. 2018, Otesbelgue et al. 2018, Ravaiano et al. 2018, Seide et al. 2018, Jacob et al. 2019a,b), reporting impairments on them. However, the only approach offering double-choice bioassays for bees was carried out by Kessler et al. (2015), exposing honeybees and bumblebees to neonicotinoids. *Melipona scutellaris* Latreille (Apidae: Meliponini) is a Brazilian native pollinator that is on the list of priority species for investigation on risk assessments (Pires and Torezani 2018) and on the list of endangered species (Ministry of the Environment). Thus, this species is interesting to study in this approach.

According to Godfray et al. (2014), pollinators may actively avoid food contaminated by insecticides or even dilute through foraging from multiple other sources. As consequence, the author stated responses at the colony or population level may mitigate or exacerbate the loss or impairment of individual insects. Thompson et al. (2015) found a direct antifeedant effect of imidacloprid and clothianidin in individual bumblebees but highlighted that this may be a compound-specific effect. On the other hand, Kessler et al. (2015) evidenced that bees cannot taste or avoid neonicotinoid pesticides.

Using this approach, assuming bees may forage naturally in pesticide-contaminated or noncontaminated areas, we aimed to investigate whether water supply interferes with the choice of neonicotinoid-dosed or nondosed food by *M. scutellaris* stingless bees and on mortality rates.

## Material and Methods

### Place and Species Studied

The tests were performed at the State University Paulista ‘Júlio de Mesquita Filho’ (UNESP), Rio Claro campus, and the species chosen as the study model was *M. scutellaris*.

### Imidacloprid Diet

Concentrations were based on the field recommendations for imidacloprid on citrus crops, registered in the ‘Ministry of Agriculture, Livestock and Supply’ (AGROFIT 2020). For dilutions, we used the active ingredient imidacloprid (Pestanal, analytic standard, Sigma–Aldrich). To estimate the amount of residue in nectar, the recommended concentration for the field was plotted in the Bee Rex table proposed by the US Environmental Protection Agency. We also consulted the imidacloprid realistic concentrations reported in nectar from sunflowers (Schmuck et al. 2001, Decourtye et al. 2003) and from oilseed rape (Bonmatin et al. 2005), which range between 0.0007 and 0.01 ng active ingredient (a.i.)/µl of nectar.

Thus, an imidacloprid stock solution was prepared at 1,000 ng a.i./µl acetone, and subsequently, the following dilutions were made to obtain the concentrations: 0.089, 0.0089, and 0.00089 (field concentration) ng a.i./µl.

### Double-Choice Food Bioassays

We carried out two bioassays: one with water supply and other without water supply. We used plastic boxes holed with needles (for the air circulation) to cage the bees, and microtubes inserted in the boxes (through a hole in the lid) to feed them.

Foragers from three nonparental colonies were grouped into sets of 10 individuals from the same colony/cage. Each cage contained three microtubes (one always containing water), weighed before and after 24 h of food supply: two with pure syrup (control: C); one with pure syrup and one with syrup containing one of the imidacloprid concentrations. Herein, the terms ‘pure syrup’ and ‘nondosed food’ have the same meaning.

The experiment was repeated under the same conditions but without water supply, i.e., only with two microtubes per cage: two with pure syrup, one with pure syrup and one with syrup containing one of the imidacloprid concentrations. Worker mortality was recorded after 24 h. We performed three replicates (30 individuals/concentration).

### Statistical Analysis

To check the normality of the data, they were submitted to the Shapiro–Wilk test. Syrup consumption between feeders in each group per experiment and water consumption between groups were analyzed by the analysis of variance (ANOVA) one-way test. Consumption between the same concentrations between the two bioassays (with and without water) was verified by the Kruskal–Wallis test. The relationship between the number of dead and alive bees between the different groups in each experiment and between the same concentrations between the bioassays (with and without water) was verified by the χ ^2^ test.

## Results

### Food Consumption

**Fig. 1.**
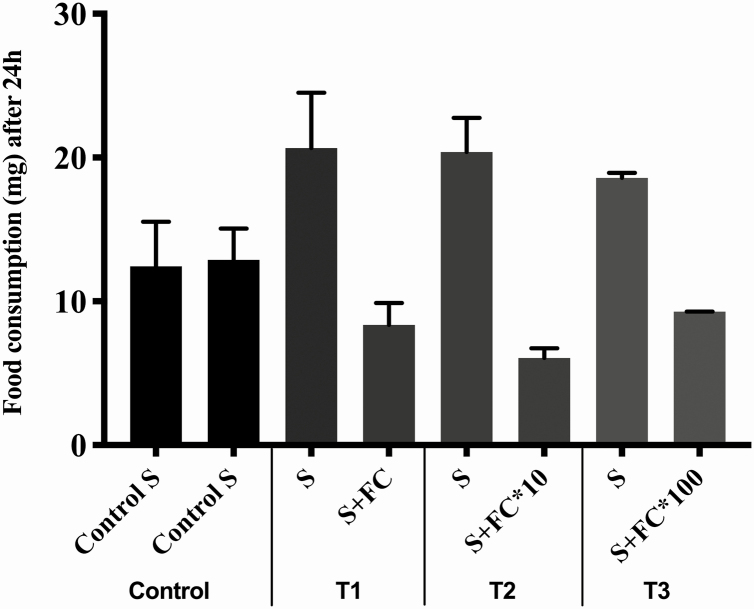
Food consumption of *Melipona scutellaris* workers between the feeders in the bioassays with water supply. Columns with the same colors indicate two feeders inside the same cage. S, syrup; FC, field concentration; Control S, feeder containing pure syrup; S, feeder containing pure syrup; S+FC, syrup containing the field concentration; S+FC*10, syrup containing the field concentration multiplied by 10; S+FC*100, syrup containing the field concentration multiplied by 100.

In the water-supply bioassays, *M. scutellaris* workers consumed significantly more nondosed food than contaminated food at the two highest concentrations: ANOVA (*F* = 6.1, *P* < 0.05; Fig. 1). When there was no water supply, the workers consumed the same amount of contaminated and uncontaminated food in all treatments: ANOVA (*F* = 0.42; *P* > 0.5). Comparison between the feeders (with the same concentrations) of the two bioassays showed no difference in food consumption between them (Kruskal–Wallis: *H* = 21.8, *P* > 0.5).

### Mortality

**Fig. 2.**
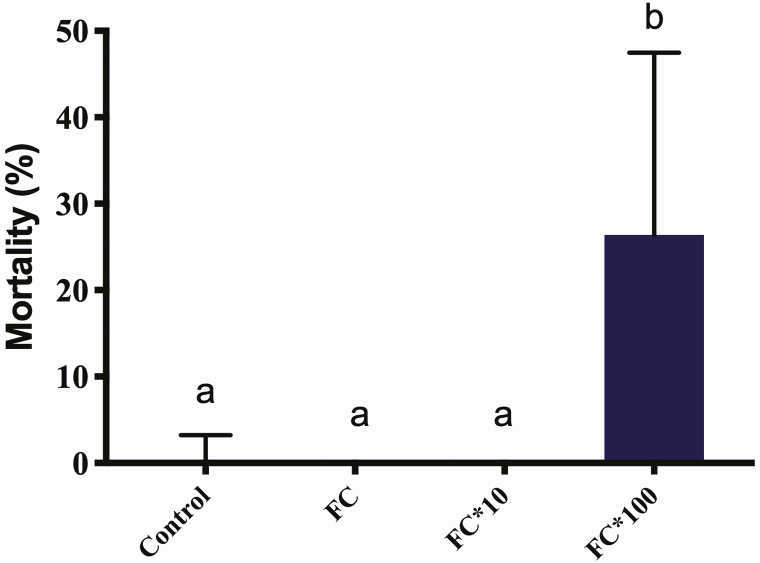
Mortality of *Melipona scutellaris* workers among the groups in the bioassay with no water supply. Control, feeder containing pure syrup; FC, syrup containing the field concentration; FC*10, syrup containing the field concentration multiplied by 10; FC*100, syrup containing the field concentration multiplied by 100. Different letters indicate significant differences.

**Fig. 3.**
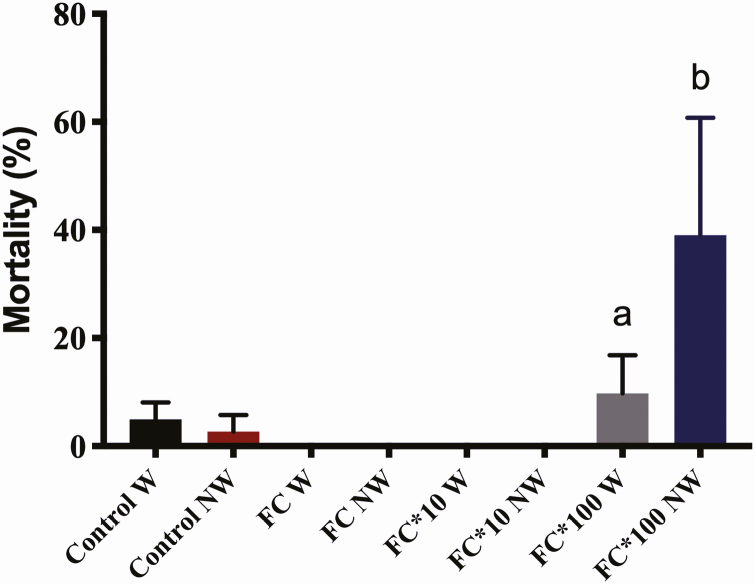
Mortality of *Melipona scutellaris* workers between the same concentrations between the different bioassays (with and without water). W, cages containing water supply; NW, cages with no water supply; control, cage containing pure syrup; FC, cage containing dosed syrup at field concentration; FC*10, syrup containing dosed syrup at field concentration multiplied by 10; FC*100, syrup containing dosed syrup at field concentration multiplied by 100. Different letters indicate significant differences.

Chi-square analysis indicated that mortality in the water-supply bioassay did not differ among the groups (*P* < 0.05). On the other hand, the same analysis between the groups in the bioassay with no water supply showed the highest concentration with the highest mortality rate in relation to all other groups (*P* < 0.0005; Fig. 2). The evaluation of the same concentrations between different bioassays (with and without water) at the highest concentration indicated a significant increase in mortality when there was no water supply (*P* < 0.005; Fig. 3).

## Discussion

Our work represents the first approach offering a double-choice opportunity for feeding in a native stingless bee, considering the water supply as an alternative to dilute the active ingredient ingested and/or to help bees to discern contaminated food and choose pure syrup. Stingless bees collect water mainly to dilute honey (Roubik 2006) and to compose larval food; this resource represents approximately 40–60% of its composition (Hartfelder and Engels 1989).

We focused our laboratory tests on realistic field concentration since it is essential for predicting a realistic field scenario (Blacquière et al. 2012). Although there is some criticism of tests using extrapolated doses/concentrations (Carreck and Ratnieks 2014), we considered using concentrations 10 and 100 times higher than our field realistic ones because in Brazil, several cases of bee mortality have been linked to the incorrect use of pesticides (MAP 2017). Thus, the excessive use of pesticides, as well as their use out of application standards, may severely compromise bees.

The opportunity to ingest water concomitantly with the double-choice test of contaminated versus uncontaminated food allowed *M. scutellaris* workers to consume significantly less dosed food at the two highest concentrations than pure food. Imidacloprid has antifeedant properties to target organisms (Nauen 1995), and at concentrations above 0.04 ng a.i./µl, imidacloprid may have repellent effects on insects (DEFRA 2007). It represents approximately half of our highest extrapolated field concentration (0.089 ng a.i./µl), which might explain justify the avoidance of ingestion by bees in the water-supply bioassays. However, with no water supply, bees showed no distinction between the feeders at all concentrations, and the same occurred when we compared the same concentrations between the two bioassays. Therefore, we proposed two assumptions: the first may corroborate the hypothesis of Godfray et al. (2014) about pesticide dilution when water is offered. Thus, the water may have helped bees taste and avoid dosed food at both the highest and intermediate concentrations (0.089 and 0.0089 ng a.i./µl, respectively). The second may be related to the water requirements of the colonies: since this resource is required by both honey dilution and larval food composition inside the nests, we may infer that the bees consumed syrup with no distinction from both feeders to supply these water requirements.

An essential point is that, at the field concentration in both bioassays, the bees consumed the same amount of food offered in the feeders, inferring in the field they are not able to differentiate between contaminated and noncontaminated food, even with water supply. Extrapolating this result to a field scenario could represent serious risks to stingless bees because the foragers are not able to detect the presence of the pesticide. Moreover, even at residual concentrations, several works reported significant sublethal effects of neonicotinoids on stingless bees, such as decreased size of newly emerged workers (de Souza Rosa et al. 2015), damaged queen selection process (Otesbelgue et al. 2018), and reduced locomotion activity of the bees (Jacob et al. 2019b). Furthermore, tests carried out by Kessler et al. (2015), with water supply, showed honeybees and bumblebees consumed more sucrose solutions laced with neonicotinoids than sucrose alone, presenting a sizeable hazard to foraging bees.

Regarding mortality, in the water-supply bioassay, the rates showed no differences among all the treatments. However, with no water supply, the highest concentration showed significantly more dead bees than other bees. Moreover, comparing the same concentrations between the two bioassays at the highest concentration, the mortality rate decreased with water supply.

Thus, from our first investigation about double-choice food, it seems that water plays an important role when bees are foraging in agricultural areas treated with neonicotinoid insecticides. However, we highlight the importance of future studies considering only-choice bioassays, i.e., with food contaminated with and without water supply, to verify the significance of water on the survival of bees and their possible detoxification capacity by cellular and molecular assessments.

## References

[CIT0001] Bernardes R. C., Barbosa W. F., Martins G. F., and Lima M. A. P.. 2018 The reduced-risk insecticide azadirachtin poses a toxicological hazard to stingless bee *Partamona helleri* (Friese, 1900) queens. Chemosphere 201: 550–556. 29533804 10.1016/j.chemosphere.2018.03.030

[CIT0002] Blacquière T., Smagghe G., van Gestel C. A., and Mommaerts V.. 2012 Neonicotinoids in bees: a review on concentrations, side-effects and risk assessment. Ecotoxicology 21: 973–992. 22350105 10.1007/s10646-012-0863-x PMC3338325

[CIT0003] Boff S., Friedel A., Mussury R. M., Lenis P. R., and Raizer J.. 2018 Changes in social behavior are induced by pesticide ingestion in a Neotropical stingless bee. Ecotoxicol. Environ. Saf. 164: 548–553. 30149353 10.1016/j.ecoenv.2018.08.061

[CIT0004] Bonmatin J. M., Marchand P. A., Charvet R., Moineau I., Bengsch E. R., and Colin M. E.. 2005 Quantification of imidacloprid uptake in maize crops. J. Agric. Food Chem. 53: 5336–5341. 15969515 10.1021/jf0479362

[CIT0005] Carreck N. L., and Ratnieks F. L.. 2014 The dose makes the poison: have ‘field realistic’ rates of exposure of bees to neonicotinoid insecticides been overestimated in laboratory studies? J. Apic. Res. 53: 607–614.

[CIT0006] Cham K. O., Nocelli R. C. F., Borges L. O., Viana-Silva F. E. C., Tonelli C. A. M., Malaspina O., Menezes C., Rosa-Fontana A. S., Blochtein B., Freitas B. M., et al 2019 Pesticide exposure assessment paradigm for stingless bees. Environ. Entomol. 48: 36–48. 30508180 10.1093/ee/nvy137

[CIT0007] Cresswell J. E., Page C. J., Uygun M. B., Holmbergh M., Li Y., Wheeler J. G., Laycock I., Pook C. J., de Ibarra N. H., Smirnoff N., et al 2012 Differential sensitivity of honey bees and bumble bees to a dietary insecticide (imidacloprid). Zoology (Jena) 115: 365–371. 23044068 10.1016/j.zool.2012.05.003

[CIT0008] de Morais C. R., Travençolo B. A. N., Carvalho S. M., Beletti M. E., Vieira Santos V. S., Campos C. F., de Campos Júnior E. O., Pereira B. B., Carvalho Naves M. P., de Rezende A. A. A., et al 2018 Ecotoxicological effects of the insecticide fipronil in Brazilian native stingless bees *Melipona scutellaris* (Apidae: Meliponini). Chemosphere 206: 632–642. 29778941 10.1016/j.chemosphere.2018.04.153

[CIT0009] de Souza Rosa A., I’Anson Price R., Ferreira Caliman M. J., Pereira Queiroz E., Blochtein B., Sílvia Soares Pires C., and Imperatriz-Fonseca V. L.. 2015 The stingless bee species, *Scaptotrigona aff. depilis*, as a potential indicator of environmental pesticide contamination. Environ. Toxicol. Chem. 34: 1851–1853. 26190578 10.1002/etc.2998

[CIT0010] Decourtye A., Lacassie E., and Pham-Delègue M. H.. 2003 Learning performances of honeybees (*Apis mellifera* L) are differentially affected by imidacloprid according to the season. Pest Manag. Sci. 59: 269–278. 12639043 10.1002/ps.631

[CIT0011] Decourtye A., Armengaud C., Renou M., Devillers J., Cluzeau S., Gauthier M., and Pham-Delègue M. H.. 2004a Imidacloprid impairs memory and brain metabolism in the honeybee (*Apis mellifera* L.). Pestic. Biochem. Physiol. 78: 83–92.

[CIT0012] Decourtye A., Devillers J., Cluzeau S., Charreton M., and Pham-Delègue M. H.. 2004b Effects of imidacloprid and deltamethrin on associative learning in honeybees under semi-field and laboratory conditions. Ecotoxicol. Environ. Saf. 57: 410–419. 15041263 10.1016/j.ecoenv.2003.08.001

[CIT0013] (DEFRA) Department for Environment, Food and Rural Affairs 2007 Assessment of the risk posed to honeybees by systemic pesticides. Project No. PS2322. DEFRA, London, United Kingdom.

[CIT0014] Feltham H., Park K., and Goulson D.. 2014 Field realistic doses of pesticide imidacloprid reduce bumblebee pollen foraging efficiency. Ecotoxicology 23: 317–323. 24448674 10.1007/s10646-014-1189-7

[CIT0015] Gill R. J., Ramos-Rodriguez O., and Raine N. E.. 2012 Combined pesticide exposure severely affects individual- and colony-level traits in bees. Nature 491: 105–108. 23086150 10.1038/nature11585 PMC3495159

[CIT0016] Godfray H. C. J., Blacquiere T., Field L. M., Hails R. S., Petrokofsky G., Potts S. G., Raine N. E., Vanbergen A. J., and McLean A. R.. 2014 A restatement of the natural science evidence base concerning neonicotinoid insecticides and insect pollinators. Proc. R. Soc. B Biol. Sci. 281: 20140558. 10.1098/rspb.2014.0558 PMC4046413 24850927

[CIT0017] Goulson D 2013 An overview of the environmental risks posed by neonicotinoid insecticides. J. Appl. Ecol. 50: 977–987.

[CIT0018] Hartfelder K., and Engels W.. 1989 The composition of larval food in stingless bees: evaluating nutritional balance by chemosystematic methods. Insectes Soc. 36: 1–14.

[CIT0019] Henry M., Béguin M., Requier F., Rollin O., Odoux J. F., Aupinel P., Aptel J., Tchamitchian S., and Decourtye A.. 2012 A common pesticide decreases foraging success and survival in honey bees. Science 336: 348–350. 22461498 10.1126/science.1215039

[CIT0020] Hladik M. L., Vandever M., and Smalling K. L.. 2016 Exposure of native bees foraging in an agricultural landscape to current-use pesticides. Sci. Total Environ. 542: 469–477. 26520270 10.1016/j.scitotenv.2015.10.077

[CIT0021] Jacob C. R. O., Malaquias J. B., Zanardi O. Z., Silva C. A. S., Jacob J. F. O., and Yamamoto P. T.. 2019a Oral acute toxicity and impact of neonicotinoids on Apis mellifera L. and *Scaptotrigona postica* Latreille (Hymenoptera: Apidae). Ecotoxicology 28: 744–753. 31254187 10.1007/s10646-019-02070-w

[CIT0022] Jacob C. R. O., Zanardi O. Z., Malaquias J. B., Souza Silva C. A., and Yamamoto P. T.. 2019b The impact of four widely used neonicotinoid insecticides on *Tetragonisca angustula* (Latreille) (Hymenoptera: Apidae). Chemosphere 224: 65–70. 30818195 10.1016/j.chemosphere.2019.02.105

[CIT0023] Kessler S., Tiedeken E. J., Simcock K. L., Derveau S., Mitchell J., Softley S., Stout J. C., and Wright G. A.. 2015 Bees prefer foods containing neonicotinoid pesticides. Nature 521: 74–76. 25901684 10.1038/nature14414 PMC4772122

[CIT0024] Laycock I., Lenthall K. M., Barratt A. T., and Cresswell J. E.. 2012 Effects of imidacloprid, a neonicotinoid pesticide, on reproduction in worker bumble bees (*Bombus terrestris*). Ecotoxicology 21: 1937–1945. 22614036 10.1007/s10646-012-0927-y

[CIT0025] Lu C., Hung Y. T., and Cheng Q.. 2020 A review of sub-lethal neonicotinoid insecticides exposure and effects on pollinators. Curr. Pollut. Rep. 6: 137–151.

[CIT0026] MAP 2017 Mapeamento de Abelhas Participativo. (https://www.gov.br/agricultura/pt-br/assuntos/camaras-setoriais-tematicas/documentos/camaras-setoriais/mel-e-produtos-das-abelhas/anos-anteriores/mapeamento-de-abelhas-participativo-map-41.pdf) (Accessed 28 September 2019).

[CIT0027] Moreira D. R., Sinópolis Gigliolli A. A., Falco J. R. P., Julio A. H. F., Volnistem E. A., Chagas F. D., Toledo V. A. A., and Ruvolo-Takasusuki M. C. C.. 2018 Toxicity and effects of the neonicotinoid thiamethoxam on *Scaptotrigona bipunctata* Lepeletier, 1836 (Hymenoptera: Apidae). Environ. Toxicol. 33: 463–475. 29377569 10.1002/tox.22533

[CIT0028] Nauen R 1995 Behaviour modifying effects of low systemic concentrations of imidacloprid on *Myzus persicae* with special reference to an antifeeding response. Pestic. Sci. 44: 145–153.

[CIT0029] Otesbelgue A., Dos Santos C. F., and Blochtein B.. 2018 Queen bee acceptance under threat: neurotoxic insecticides provoke deep damage in queen-worker relationships. Ecotoxicol. Environ. Saf. 166: 42–47. 30245292 10.1016/j.ecoenv.2018.09.048

[CIT0030] Pires C. S. S., and Torezani K. R. S.. 2018 Seleção de espécies de abelhas nativas para avaliação de risco de agrotóxicos. Ibama, Brasília, Brazil.

[CIT0031] Ravaiano S. V., Barbosa W. F., Tomé H. V. V., Campos L. A. O., and Martins G. F.. 2018 Acute and oral exposure to imidacloprid does not affect the number of circulating hemocytes in the stingless bee *Melipona quadrifasciata* post immune challenge. Pestic. Biochem. Physiol. 152: 24–28. 30497707 10.1016/j.pestbp.2018.08.002

[CIT0032] Roubik D. W 2006 Stingless bee nesting biology. Apidologie 37: 124–143.

[CIT0033] Schmuck R., Schöning R., Stork A., and Schramel O.. 2001 Risk posed to honeybees (*Apis mellifera* L, Hymenoptera) by an imidacloprid seed dressing of sunflowers. Pest Manag. Sci. 57: 225–238. 11455652 10.1002/ps.270

[CIT0034] Schneider C. W., Tautz J., Grünewald B., and Fuchs S.. 2012 RFID tracking of sublethal effects of two neonicotinoid insecticides on the foraging behavior of *Apis mellifera*. PLoS One 7: e30023. 22253863 10.1371/journal.pone.0030023 PMC3256199

[CIT0035] Seide V. E., Bernardes R. C., Pereira E. J. G., and Lima M. A. P.. 2018 Glyphosate is lethal and cry toxins alter the development of the stingless bee *Melipona quadrifasciata*. Environ. Pollut. 243: 1854–1860. 30408873 10.1016/j.envpol.2018.10.020

[CIT0036] Thompson H. M., Wilkins S., Harkin S., Milner S., and Walters K. F.. 2015 Neonicotinoids and bumblebees (*Bombus terrestris*): effects on nectar consumption in individual workers. Pest Manag. Sci. 71: 946–950. 25132051 10.1002/ps.3868

[CIT0037] Whitehorn P. R., O’Connor S., Wackers F. L., and Goulson D.. 2012 Neonicotinoid pesticide reduces bumble bee colony growth and queen production. Science 336: 351–352. 22461500 10.1126/science.1215025

